# Mitochondrial Neurogastrointestinal Encephalomyopathy: Into the Fourth Decade, What We Have Learned So Far

**DOI:** 10.3389/fgene.2018.00669

**Published:** 2018-12-21

**Authors:** Dario Pacitti, Michelle Levene, Caterina Garone, Niranjanan Nirmalananthan, Bridget E. Bax

**Affiliations:** ^1^Molecular and Clinical Sciences Research Institute, St George's, University of London, London, United Kingdom; ^2^MRC Mitochondrial Biology Unit, Cambridge Biomedical, Cambridge, United Kingdom; ^3^St George's University Hospitals NHS Foundation Trust, London, United Kingdom

**Keywords:** MNGIE, thymidine phosphorylase, mitochondrial disease, rare disease, deoxyribonucleoside, TYMP, mitochondrial DNA, mitochondrial neurogastrointestinal encephalomyopathy

## Abstract

Mitochondrial neurogastrointestinal encephalomyopathy (MNGIE) is an ultra-rare metabolic autosomal recessive disease, caused by mutations in the nuclear gene *TYMP* which encodes the enzyme thymidine phosphorylase. The resulting enzyme deficiency leads to a systemic accumulation of the deoxyribonucleosides thymidine and deoxyuridine, and ultimately mitochondrial failure due to a progressive acquisition of secondary mitochondrial DNA (mtDNA) mutations and mtDNA depletion. Clinically, MNGIE is characterized by gastrointestinal and neurological manifestations, including cachexia, gastrointestinal dysmotility, peripheral neuropathy, leukoencephalopathy, ophthalmoplegia and ptosis. The disease is progressively degenerative and leads to death at an average age of 37.6 years. As with the vast majority of rare diseases, patients with MNGIE face a number of unmet needs related to diagnostic delays, a lack of approved therapies, and non-specific clinical management. We provide here a comprehensive collation of the available knowledge of MNGIE since the disease was first described 42 years ago. This review includes symptomatology, diagnostic procedures and hurdles, *in vitro* and *in vivo* disease models that have enhanced our understanding of the disease pathology, and finally experimental therapeutic approaches under development. The ultimate aim of this review is to increase clinical awareness of MNGIE, thereby reducing diagnostic delay and improving patient access to putative treatments under investigation.

## Disease Name and Synonyms

Mitochondrial neurogastrointestinal encephalomyopathy (MNGIE, Online Mendelian inheritance in Man #603041, Genome Database accession #9835128) is a fatal inherited metabolic disorder caused by mutations in a nuclear gene controlling the metabolism of pyrimidine deoxyribonucleosides and indirectly influencing the replication and expression of the mitochondrial genome (Nishino et al., 1999; Hirano et al., 2004b). In the past, the disorder has also been referred to as:

- Congenital oculoskeletal myopathy
- Mitochondrial myopathy with sensorimotor polyneuropathy, ophthalmoplegia, and pseudo-obstruction (MEPOP)
- Mitochondrial neurogastrointestinal encephalopathy syndrome
- Myoneurogastrointestinal encephalopathy syndrome
- Chronic intestinal pseudo-obstruction with myopathy and ophthalmoplegia
- Polyneuropathy, ophthalmoplegia, leukoencephalopathy and intestinal pseudo-obstruction (POLIP);
- Oculogastrointestinal encephalopathy syndrome; Oculogastrointestinal muscular distrophy (OGIDM)
- Thymidine phosphorylase deficiency

## History

The condition was first described in 1976 by Okamura et al., who reported a 22-year old cachectic man experiencing ptosis, ophthalmoplegia, dysphagia and myopathy. Histological findings revealed mitochondrial abnormalities in skeletal muscles and liver cells. The authors recognized that the condition exhibited familial tendencies and therefore proposed the term congenital oculoskeletal myopathy to describe the disorder (Okamura et al., 1976). Analogous patients with ocular, neurological, skeletal, and gastrointestinal involvement were additionally described in the literature, and Bardosi et al. also reported leukoencephalopathy in a patient with a history of extraocular and skeletal myopathy and gastrointestinal symptoms (Anuras et al., 1983; Ionasescu, 1983; Ionasescu et al., 1983, 1984; Bardosi et al., 1987; Faber et al., 1987; Simon et al., 1990). In 1994, Hirano et al. conducted a systematic review of all reported cases of the condition and proposed the current nomenclature mitochondrial neurogastrointestinal encephalomyopathy (MNGIE), which highlighted the central features of this mitochondrial disorder (Hirano et al., 1994). The etiology was only elucidated in 1999, when the condition was attributed to a deficiency in thymidine phosphorylase, E.C.2.4.2.4 (Nishino et al., 1999).

## Molecular Etiology

**Figure 1:**
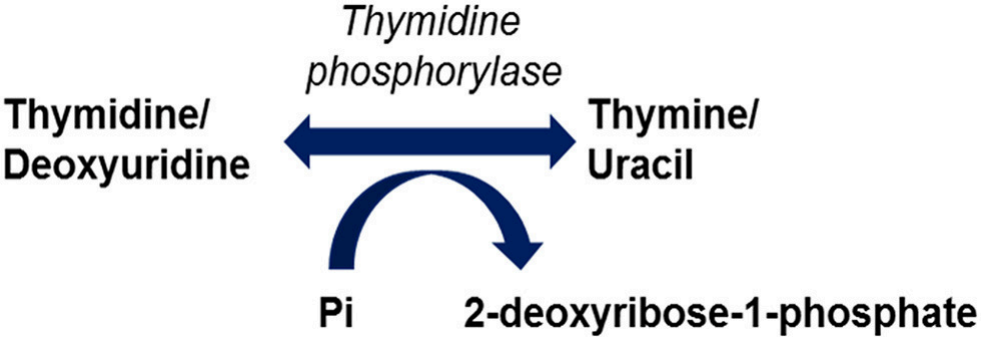
Reactions catalysed by thymidine phosphorylase.

Mutations in the *TYMP* gene and a subsequent deficiency in thymidine phosphorylase activity are the causative factors in the pathogenesis of MNGIE. Thymidine phosphorylase is also referred to as gliostatin and platelet derived-endothelial cell growth factor (PD-ECGF). Structurally the peptide is composed of two subunit homodimers each with a molecular weight of ~50 kilodaltons (Norman et al., 2004). Thymidine phosphorylase catalyses the reversible phosphorylation of thymidine (also known as deoxythymidine) and deoxyuridine to 2-deoxyribose 1-phosphate and their respective bases, thymine and uracil, Figure 1 (Nishino et al., 1999). Thymidine phosphorylase has a pivotal role in the nucleoside salvage metabolic pathway, and in the recycling of pyrimidine bases by regulating the availability of thymidine for DNA biosynthesis (Nishino et al., 1999; Levene et al., 2013).

**Figure 2:**
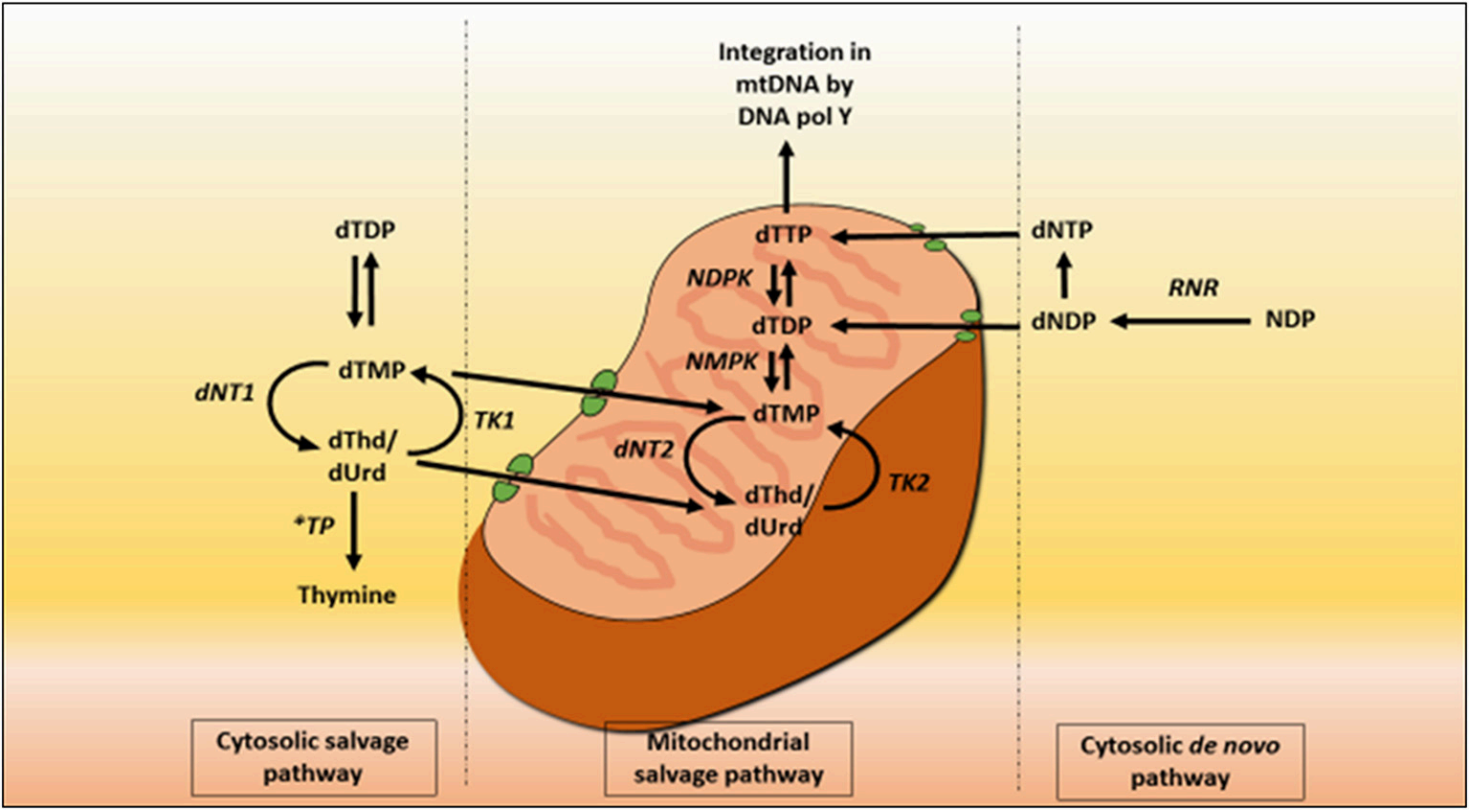
Deoxynucleotide salvage and *de-novo* synthesis pathways. Abbreviations are as follows: deoxythymidine (dThd), deoxyuridine (dUrd), deoxythymidine monophosphate (dTMP), deoxythymidine diphosphate (dTDP), deoxynucleotidase 1 (dNT1), thymidine phosphorylase (TP), thymidine kinase 1 (TK1), deoxynucleotidase 2 (dNT2), nucleotide monophosphate kinase (NMPK), nucleotide diphosphate kinase (NDPK), deoxythymidine triphosphate (dTTP), thymidine kinase 2 (TK2), DNA polymerase Y (DNA pol Y), nucleotide diphosphate (NDP), ribonucleotide reductase (RNR), deoxyribonucleotide diphosphate (dNDP), and deoxynucleotide triphosphate (dNTP).

Mitochondrial deoxyribonucleoside pools are maintained by both the cytoplasmic *de novo* pathway and the salvage pathway located within the mitochondrion, Figure 2. In proliferating cells, the major source of mitochondrial deoxyribonucleotide diphosphates originates from the cytoplasmic *de novo* pathway, whereby a transporter located in the mitochondrial membrane transports the deoxyribonucleotide triphosphates (dNTPs) synthesized in the cytosol into the mitochondrial matrix for the synthesis of mtDNA. In quiescent cells (such as muscles and neurons) the cytoplasmic *de novo* pathway is no longer required for nuclear DNA replication and is thus down-regulated due to a reduction in ribonucleotide reductase activity, leading to a marked reduction in cytosolic dNTP pools (Rötig and Poulton, 2009). mtDNA synthesis is not limited to the S-phase of the cell cycle and mitochondria are continuously replicating, even in post-mitotic cells. Therefore, a constant supply of nucleotides is essential for the maintenance of the mitochondrial genome and hence the salvage pathway becomes important. The loss of function of thymidine phosphorylase leads to an enhancement of thymidine salvage through the action of thymidine kinase 2 (TK2) which is constitutively expressed in the mitochondria. Of note, thymidine kinase 1 (TK1) is upregulated only in proliferating cells. TK2 converts thymidine to thymidine monophosphate, as well as deoxyuridine and deoxycytidine to their respective monophosphate nucleotides, and is therefore believed to contribute to the generation of the deoxynucleotide pool imbalances in the mitochondria (Nishino et al., 1999; Hirano et al., 2004b).

**Figure 3:**
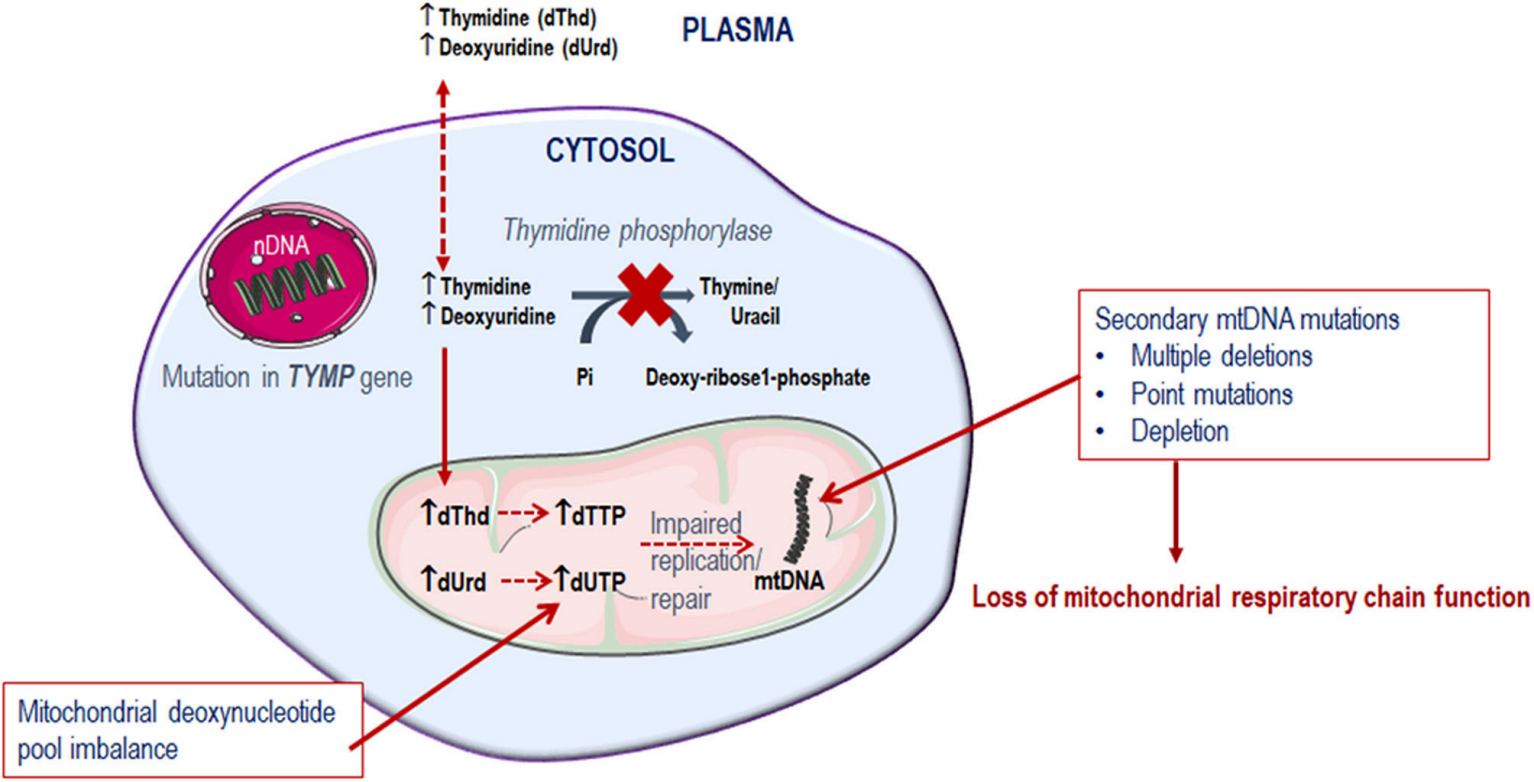
Metabolic defect in MNGIE.

Since thymidine phosphorylase is crucial in the pyrimidine metabolic pathway for the catabolism of thymidine, its dysfunction compromises the deoxyribonucleoside pool balance. It is observed that the tissues affected in MNGIE are predominantly post-mitotic (Samsonoff et al., 1997; Nishino et al., 1999; Pontarin et al., 2006; Zhou et al., 2008; Balasubramaniam et al., 2014). Consequently, because of the deoxyribonucleoside pool imbalance, combined with the limited ability of the mitochondrial DNA polymerase γ to repair DNA, mtDNA gradually accumulates mutations over time, which ultimately leads to the failure of mitochondria to perform oxidative phosphorylation, Figure 3 (Bogenhagen, 1999; Nishigaki et al., 2004).

In MNGIE, phenotypic manifestations of the disease develop when a threshold level of mutant mtDNA is reached, which is generally when more than 80–90% of total mitochondria are affected (Mazat et al., 2001; Nishigaki et al., 2003). This threshold effect and the heteroplasmic nature of mitochondria (the existence of two or more mitochondrial genotypes within the same cell) very likely account for the protracted interval before the condition manifests and contributes to the heterogeneous phenotypes observed.

In humans thymidine phosphorylase is abundantly expressed in blood cells (platelets, macrophages, peripheral lymphocytes, stromal cells, and reticulocytes), liver, lungs, brain and tissues of the digestive tract; however it is not expressed in skeletal muscle, kidneys or adipose tissue (Fox et al., 1995). In addition to its enzymatic activity driving the salvage pathway, thymidine phosphorylase also functions as a signaling molecule playing an essential role in a number of processes (Li and Yue, 2017). Thymidine phosphorylase acts as a growth factor with strong pro-angiogenic effects and is a potent mitogen for endothelial cells (Miyazono et al., 1987; O'Brien et al., 1996). In addition it has been demonstrated that thymidine phosphorylase is an inhibitor of apoptosis (Li and Yue, 2017). Platelets are a major source of thymidine phosphorylase, and it has been shown that the protein is involved in platelet activation through exhibiting a potent pro-thrombotic effect (Miyazono et al., 1987; Li and Yue, 2017). Moreover, thymidine phosphorylase shows a strong inhibitory effect on all glial cells and has been demonstrated to exert a neurotrophic effect on cortical neurons (Asai et al., 1992a,b; Ueki et al., 1993).

A deficiency in enzymatic activity (<5% of healthy individuals) results in elevated concentrations of thymidine and deoxyuridine in tissues and body fluids, which consequently generate deoxyribonucleoside pool imbalances, leading to impaired mtDNA replication, and ultimately mitochondrial failure (Hirano et al., 1994; Spinazzola et al., 2002; Martí et al., 2003; Valentino et al., 2007). In patients with MNGIE, deoxyribonucleoside concentrations can reach plasma levels of 3.9–17.7 μmol/L for thymidine and 5.5–24.4 μmol/L for deoxyuridine, compared to undetectable levels in healthy unaffected individuals (Hirano et al., 1998; Martí et al., 2003). In tissues such as the small intestine, kidney, liver, peripheral nerve, and occipital white matter, levels in the range of 38–1532 nmoles/g protein for thymidine and 32–728 nmoles/g protein for deoxyuridine have been reported (Valentino et al., 2007). Thymidine and deoxyuridine are ultra-filterable, and thus the systemic accumulation of thymidine and deoxyuridine is further exacerbated by the efficiency of renal reabsorption of these deoxyribonucleosides (Okamura et al., 1976; Hirano et al., 1998; Spinazzola et al., 2002; Garone et al., 2011).

### Disease-Causing Mutations

#### *TYMP* Mutations

**Figure 4:**
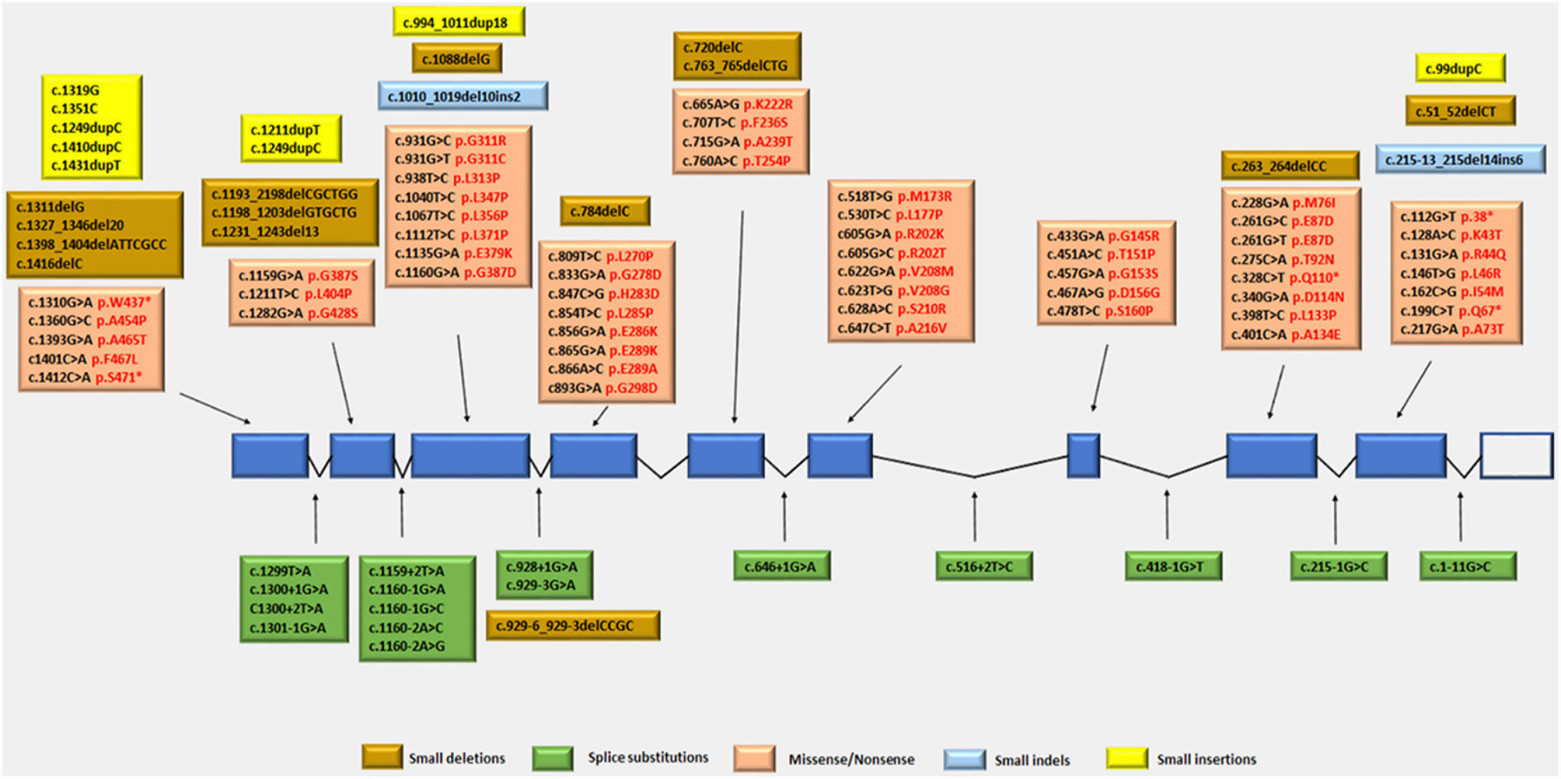
Pathogenic *TYMP* gene mutations (NM_001113755.2; NP_001107227) in exonic and intronic regions. Protein changes, where known are indicated in red font.

The *TYMP* gene has been mapped to the chromosomal locus 22q13.32-qter (Hirano et al., 1998; Nishino et al., 1999, 2000). Since the identification of *TYMP* as the gene responsible for MNGIE, 92 different mutations have been reported by the Human Gene Mutation Database (HGMD Professional 2018.2, accessed September 2018) (Stenson et al., 2014), including 56 missense/nonsense (Nishino et al., 1999, 2000; Gamez et al., 2002; Kocaefe et al., 2003; Hirano et al., 2004b; Martín et al., 2004; Marti et al., 2005; Said et al., 2005; Slama et al., 2005; Carod-Artal et al., 2007; Schupbach et al., 2007; Monroy et al., 2008; Massa et al., 2009; Poulton et al., 2009; Bariş et al., 2010; Garone et al., 2011; Nalini and Gayathri, 2011; Scarpelli et al., 2012; Mihaylova et al., 2013; Suh et al., 2013; Benureau et al., 2014; Vondrácková et al., 2014; Peedikayil et al., 2015; Wang et al., 2015; Karyampudi et al., 2016), 13 splice site mutations (Nishino et al., 1999, 2000; Kocaefe et al., 2003; Szigeti et al., 2004b; Slama et al., 2005; Laforce et al., 2009; Taanman et al., 2009; Garone et al., 2011; Libernini et al., 2012; Halter et al., 2015), 13 small deletions (Nishino et al., 1999, 2000; Blazquez et al., 2005; Slama et al., 2005; Poulton et al., 2009; Filosto et al., 2011; Garone et al., 2011; Torres-Torronteras et al., 2011; Halter et al., 2015; Karyampudi et al., 2016), 6 small insertions (Nishino et al., 1999; Gamez et al., 2002; Hirano et al., 2004b; Kintarak et al., 2007; Poulton et al., 2009; Cardaioli et al., 2010), 2 small indels (Garone et al., 2011; Libernini et al., 2012) 1 gross insertion (Wang et al., 2017) and 1 gross deletion (Vondrácková et al., 2014). These mutations have been mapped to either exonic or intronic regions, with some identified as benign and some as pathogenic variants. Figure 4 summarizes the known pathogenic variants associated with MNGIE, based on their classification and location on the *TYMP* gene.

The mutation distribution suggests founder effects for some mutations such as c.866A>G in Europeans and c. 518T>G in individual from the Dominican Republic (Garone et al., 2011).

#### Effect on Mitochondrial DNA

The secondary mtDNA mutations reported in MNGIE, are caused by the toxic accumulations of thymidine and deoxyuridine, because of the nuclear *TYMP* mutations. These secondary mutations have been identified as mtDNA deletions, depletion and misincorporations. Acquired secondary mitochondrial mutations appear to be conserved in most cases, with 86% of detected mutations being T>C transitions preceded by a short run of As. This can be explained by a competition between guanosine monophosphate (GMP) and adenosine monophosphate (AMP) for incorporation opposite to a thymine residue on the template DNA. After the occurrence of misincorporations, elevated thymidine triphosphate (TTP) levels accelerate polymerase γ exonuclease removal of mismatches, so that the T is switched to C during mtDNA replication; these mutations ultimately lead to failure of oxidative phosphorylation (Nishigaki et al., 2003). Certain mtDNA genes appear to be hotspots for mutations in MNGIE, such as the *ND5* gene which is prone to multiple deletions (Nishigaki et al., 2004). Gonzalez-Vioque et al. proposed a hypothesis for the mtDNA depletion observed in MNGIE, suggesting that mitochondrial replication is not affected by the accumulation of nucleosides *per se*, but rather by the secondary depletion of deoxycytidine stemming from an increase in TTP pools, thus limiting its availability for mtDNA biosynthesis (González-Vioque et al., 2011).

## Epidemiology

MNGIE is an ultra-rare disorder with a European incidence of <1 in a million, with Orphanet estimating the prevalence to be 1–9 in 1,000,000 world-wide (Orphanet, 2018). Estimated epidemiological data is largely confined to various case reports or case series from several groups over the last two decades. Halter et al. (2010), quotes a personal communication from M. Hirano of fewer than 200 identified patients world-wide (Halter et al., 2010). In the only systematic study of epidemiology of the disease, a minimum prevalence estimate of ~0.15 per 1,000,000 was established in a prospective Italian survey in the Emilia-Romagna region (D'Angelo et al., 2016).

MNGIE is distributed amongst a widely distributed and ethnically diverse population including Hispanics, Americans, Western Europeans, Jamaicans, Ashkenazi Jewish, Middle Eastern, and Canadians (Nishino et al., 2001; Hirano et al., 2004b; Kintarak et al., 2007; Borhani Haghighi et al., 2009; Bariş et al., 2010). An ethnic predisposition has yet to be established. However since the pathology is inherited in an autosomal recessive fashion, populations in which consanguineous relationships are common are more at risk (Walia et al., 2006).

It is currently not possible to be confident about stating the prevalence of MNGIE as the disorder is appreciably under-diagnosed due its multisystem presentation and rarity (Filosto et al., 2011; Scarpelli et al., 2012). The condition is not familiar to a majority of clinicians, and patients typically undergo referral to several different specialities over a protracted period of time before a diagnosis is achieved. The diagnosis is often not made until after the death of one or two family members with similar symptomatology.

## Clinical Description

**Figure 5:**
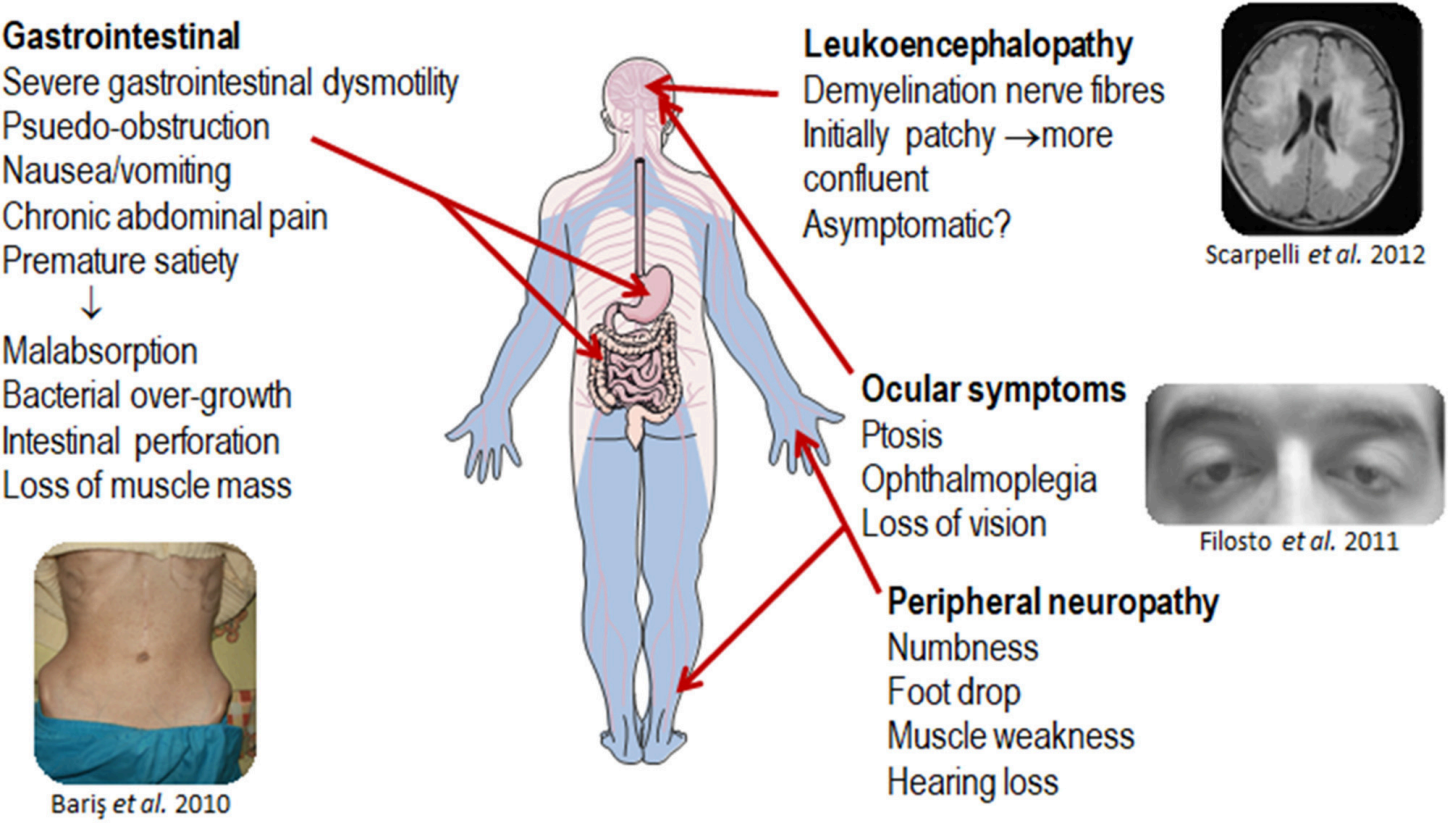
Major clinical features of MNGIE. Copyright permission was obtained for the reproduction of images taken from Bariş et al. (2010), Filosto et al. (2011), Scarpelli et al. (2012).

MNGIE is a relentlessly progressive and degenerative disease, causing significant morbidity. Although the clinical presentation of MNGIE is homogeneous, it is characterized by a complex clinical picture, with the involvement of multiple organ systems to differing extents in different individuals, Table 1. The mean age mortality of 37.5 years (Nishino et al., 2000). Based on a review of the literature, we propose a classification of the major and minor clinical features of MNGIE. The major clinical features for the diagnosis of MNGIE are severe gastrointestinal dysmotility, cachexia, peripheral neuropathy, ocular symptoms, and asymptomatic diffuse leukoencephalopathy, Figure 5 (Hirano et al., 1994, 2004b; Nishino et al., 2000). Other signs and symptoms represent a minor clinical criterion for the diagnosis of the disease, including certain neurological, muscular, cardiac and endocrine features, as well as other sporadic manifestations discussed below.

**Table 1:** List of clinical features reported in MNGIE.

**Features**	**Sign/symptom**	**Frequency**
Neurological	Peripheral neuropathy	+++
	Hearing loss	++
	Leukoencephalopathy	+++
	Seizures	+
	Migraine	+
	Anxiety	+
	Depression	+
	Cognitive dysfunction	+
	Dementia	+
	Mental retardation	+
	Memory loss	+
	Ataxia	+
	Trigeminal neuralgia	+
Neuro-ophthalmic	Ophthalmoplegia	+++
	Ophthalmoparesis	+++
	Ptosis	+++
	Glaucoma	+
	Pigmentary retinopathy	+
Muscular	Myopathy	++
	Red ragged fibers	++
Gastrointestinal	Intestinal pseudo-obstruction	++
	Constipation	++
	Abdominal cramps	++
	Nausea	+++
	Emesis	+++
	Borborygmy	++
	Diarrhoea	++
	Dysphagia	+++
	Gastroparesis	+++
	Cachexia	+++
	Weight loss	+++
	Oesophageal varices	++
	Megacolon	+
	Diverticulosis	++
	Intestinal perforation	++
	Peritonitis	++
	Hepatic steatosis	++
	Hepatomegaly	+
	Cirrhosis	+
Endocrine/Metabolic	Diabetes	++
	Hyperlipidaemia	++
	Hypertriglyceridemia	++
	Hypergonadotropic hypogonadism	+
Cardiac	Long QT	+
	Supraventricular tachycardia	+
	Ventricular hypertrophy	+
	Mitral valve prolapse	+
Reproductive	Ovarian failure	+
	Erectile dysfunction	+
	Amenorrhea	+
Haematological	Anaemia	+
Dermatological	Psoriasis	+
Developmental	Short stature	++

*+++ indicates a major diagnostic feature of MNGIE, ++ a common clinical presentation and + a sporadic feature*.

### Onset of Symptoms

The onset of MNGIE disease is usually between the first and second and decade of life, with an average age of onset at 18.5 years (Nishino et al., 2001; Garone et al., 2011); however, reported age of onset may not be accurate due to delay in diagnosis stemming from the subtlety of non-specific symptoms (Hirano et al., 1998). A few cases of late onset beyond the third decade and as late as the fifth decade have been reported, which were associated with compound heterozygous *TYMP* mutations and a less severe phenotype characterized by a partial reduction of thymidine phosphorylase activity (Marti et al., 2005; Massa et al., 2009). The earliest reported age of onset is five months of age (Garone et al., 2011). However, in a majority of patients, the first insidious symptoms manifest during childhood (Garone et al., 2011).

### Major Clinical Criteria for Diagnosis

#### Gastrointestinal Features

Gastrointestinal dysmotility is one of the most common features of MNGIE, with patients manifesting intestinal pseudo-obstruction, abdominal cramps, enteric bacteria overgrowth, nausea, vomiting, borborygmy, diarrhea, dysphagia, and gastroparesis (Garone et al., 2011). Gastrointestinal dysfunctions eventually lead to malnutrition, cachexia and severe weight loss with averages of 15 Kg loss being reported (Nishino et al., 1999). Regardless of the gastrointestinal irregularities, patients appear to have normal serum levels of vitamins E, B12 and folate (Holt et al., 1990; Mueller et al., 1999). Patients with MNGIE often have a frail and slender physique with reduced muscle mass. It is unclear whether the gastrointestinal involvement is the result of intestinal smooth muscle dysfunction caused by mitochondrial defects or whether damage to the enteric nervous system is primarily responsible (Verma et al., 1997). It is recognized that as the disease progresses, the gastrointestinal symptoms are exacerbated, with patients dying from severe malnutrition and gastrointestinal complications such as esophageal varices, megacolon, diverticulosis, bowel perforations, peritonitis, and bacterial overgrowth (Martinez-Garcia et al., 2001; Aksoy et al., 2005; Moran et al., 2008; Granero Castro et al., 2010; Scarpelli et al., 2012; Dreznik et al., 2014; Kalkan et al., 2015; Finsterer and Frank, 2017). Patients exhibiting hepatopathies have also been reported, including cases of hepatic steatosis, hepatomegaly, increased transaminases and cirrhosis (Schupbach et al., 2007; Garone et al., 2011; Finkenstedt et al., 2013).

#### Peripheral Neuropathy

In the peripheral nervous system, MNGIE results in neuropathy (Garone et al., 2011). This manifests as numbness, paraesthesia (tingling sensation), foot drop and limb weakness (Garone et al., 2011). The neuropathy has been shown to be demyelinating in all cases, with half the reported cases also having axonal neuropathy (Garone et al., 2011). Ultra-structurally, nerve biopsies reveal segmental demyelination, myelin sheath abnormalities, and axonal degeneration and depletion (Bedlack et al., 2004). Unilateral or bilateral foot drop and clawed hands may also be observed (Garone et al., 2011). The neuropathy is characterized by decreased motor and sensory nerve conduction velocities, prolonged F-wave latency and partial conduction block (Bedlack et al., 2004). The clinical and electrophysiological features may mimic those of other conditions including chronic inflammatory demyelinating polyradiculoneuropathy (CIDP) and Charcot-Marie-Tooth disease (Bedlack et al., 2004; Needham et al., 2007).

#### Ocular Symptoms

Ocular symptoms such as ptosis and ophthalmoplegia or ophthalmoparesis are also common neurological findings in patients with MNGIE (Barboni et al., 2004). Other uncommon ocular manifestations include reports of mild myopia, glaucomatous-like features and tilted disc with focal defects of the retinal nerve fibers (Barboni et al., 2004). Rarely, pigmentary retinopathy can also be observed in patients with MNGIE (Hirano et al., 1994; Nishino et al., 1999; Aksoy et al., 2005; Garone et al., 2011).

#### Leukoencephalopathy

One peculiarity of MNGIE is the typically paucisymptomatic central nervous system (CNS) involvement. In the majority of affected individuals, this is identified as white matter lesions that remain subclinical and visible as a signal change on Magnetic Resonance Imaging (MRI) scans indicating progressive leukoencephalopathy (Garone et al., 2011). Leukoencephalopathy is the hallmark feature of the pathology and its presence in combination with gastrointestinal and neuropathic symptoms significantly narrows the differential diagnosis to MNGIE. The leukoencephalopathy as identified by MRI, is initially patchy but progressively becomes more diffuse, appearing as hypointense on T1- and hyperintense on T2- weighted images and in fluid-attenuated inversion recovery (FLAIR) and fast spin echo (FSE) T2 sequences (Garone et al., 2011; Çoban et al., 2013; Scarpelli et al., 2013; Gramegna et al., 2018). The most involved region of the CNS in MNGIE is the subcortical white matter. Hyperintensities in the subcortical U-fibers and occasionally in the corpus callosum have been reported, alluding to problems in the interhemispheric communication (Millar et al., 2004; Scarpelli et al., 2013). Areas less frequently affected include the capsular white matter, and the white matter in the basal ganglia, thalami, midbrain, pons and cerebellum (Millar et al., 2004; Barragán-Campos et al., 2005; Scaglia et al., 2005; Petcharunpaisan and Castillo, 2010). The reasons why the leukoencephalopathy remains asymptomatic are yet to be elucidated, however it has been suggested that hyperintense lesions observed by MRI could be the result of alterations in the brain microvasculature causing vasogenic oedema and glial dysfunctions (Szigeti et al., 2004a; Scarpelli et al., 2013; Gramegna et al., 2018). Whether there are subtle neuropsychiatric or cognitive changes associated with the leukoencephalopathy remains an open question.

### Minor Clinical Criteria for Diagnosis

#### Other Central Nervous System Associated Features

A growing body of evidence suggests that the CNS involvement in MNGIE could be more symptomatic than initially described (Garone et al., 2011). For instance, in a number of patients, cases of seizures, including generalized tonic-clonic seizures, have been reported (Walia et al., 2006; Yavuz et al., 2007; Garone et al., 2011). Garone et al. indicated that six patients with MNGIE from their study cohort of 102 complained of headache, and similarly an independent study evaluating the frequency of migraine in mitochondrial diseases identified one patient with MNGIE suffering from episodes of cephalgia (Garone et al., 2011; Vollono et al., 2018). Psychiatric manifestations have been noted in MNGIE, with patients reporting anxiety and depression, although it remains unclear whether these are secondary to the psychological aspect of coping with a terminal debilitating condition (Garone et al., 2011; Scarpelli et al., 2013). Cases of patients with dementia and cognitive dysfunction have also been reported, with one patient also showing mental retardation (Hirano et al., 1994; Carod-Artal et al., 2007; Garone et al., 2011). Problems with memory, concentration and visuospatial orientation have also been observed in some patients (Borhani Haghighi et al., 2009). Ataxia is also occasionally observed in MNGIE (Hirano et al., 1994). A case study reported trigeminal neuralgia in one patient with MNGIE, and the authors suggested that this could be ascribable to demyelinating lesions in the trigeminal intrapontine fibers within the brain stem, as observed in MRI images, in an analogous way to that observed in patients with multiple sclerosis (Peker and Necmettin Pamir, 2005).

#### Sensorineural Hearing Impairment

Hearing loss is reported as one of the most common neurologic features in patients with MNGIE(Hirano et al., 2004b; Bariş et al., 2010; Cardaioli et al., 2010; Garone et al., 2011). For instance, the study by Garone et al. reported that 39% of patients, from a cohort of 102, presented with anacusis (Garone et al., 2011). Hearing loss appears to be sensorineural and is not common during the presentation of the first symptoms, however it is more prominent in the later stages of the disease (Hirano et al., 2004b).

#### Muscular Features

Thymidine phosphorylase is not physiologically expressed in skeletal muscle, but the muscle from patients with MNGIE shows alterations in mtDNA, COX–deficient and ragged red fibers and respiratory chain enzymatic defects (Yoshimura et al., 1990; Hirano et al., 1994; Nishino et al., 1999). This observation has in the past been referred to as the “muscle paradox” (Nishino et al., 1999; Hirano et al., 2004b); it is now known that the pathological involvement of this tissue is due to systemic accumulations of the pyrimidine nucleosides rather than an absence of thymidine phosphorylase activity itself (Nishino et al., 1999). In healthy individuals, the absence of detectable thymidine and deoxyuridine suggests that thymidine phosphorylase regulates intracellular and extracellular levels of these deoxyribonucleosides. It is believed that the platelets and other blood cells, as wells as tissues rich in thymidine phosphorylase activity regulate these levels, especially in those tissues which lack thymidine phosphorylase (Nishino et al., 1999, 2000; Spinazzola et al., 2002; Hirano et al., 2004a). Of note, some patients with MNGIE do not display a primary skeletal muscle involvement (Szigeti et al., 2004b; Cardaioli et al., 2010).

#### Endocrine and Metabolic Dysfunctions

Sporadically, there have been reports of MNGIE patients presenting endocrine and metabolic dysfunctions, including endocrine/exocrine pancreatic insufficiency, diabetes, amylase increases and glucose intolerance (Garone et al., 2011). Alteration in plasma lipid profiles have also been observed in patients presenting severe hyperlipidaemia and hypertriglyceridemia (Bariş et al., 2010; Garone et al., 2011). A reduction of mitochondrial function is likely to be an important contributor to the lipid accumulation and insulin resistance. Furthermore, there have been two reports of patients with MNGIE manifesting hypergonadotropic hypogonadism (Carod-Artal et al., 2007; Kalkan et al., 2012).

#### Immunodeficiency

In patients with MNGIE, gastrointestinal dysfunctions can lead to a dysbiosis of the intestinal microbiome, and current research has shown that alterations in the gut flora can impact on systemic adaptive immune responses (Round and Mazmanian, 2009; Filosto et al., 2011; van den Elsen et al., 2017). Additionally, patients often manifest complications, which include diverticular ruptures, intestinal perforations and aspiration pneumonia which expose individuals to infections that can present fatal outcomes. Recurrent infections have been reported, with these adverse events contributing to the worsening of the symptoms and prognosis (Garone et al., 2011). In one case report, a patient was described with bacterial endocarditis, suggesting the immune system may be suppressed in MNGIE (Yolcu et al., 2014).

#### Cardiac Complications

Cardiac manifestations are usually asymptomatic in MNGIE, although the study of Garone et al., reported occasional cardiac complications, including a prolonged QT interval, cardiac arrest and supraventricular tachycardia (Garone et al., 2011; El-Hattab and Scaglia, 2016). Abnormal ECG has also been reported in a number of patients, with individuals displaying left ventricular hypertrophy and bundle branch block (Hirano et al., 2004b). A study also described cardiac dysfunction in affected twins, presenting mitral valve prolapse and systolic heart murmurs (Schupbach et al., 2007). Another case study reported the death of two brothers due to cardiomyopathy (Borhani Haghighi et al., 2009).

#### Other Sporadic Features

From a review of the literature, other non-specific manifestations have been reported, which are sporadic and are not clearly attributable to MNGIE or the secondary ailments of the disease. Amongst these less common manifestations, patients have been reported with ovarian failure (Borhani Haghighi et al., 2009), anemia, amenorrhea (Gamez et al., 2002; Garone et al., 2011) and psoriasis (Garone et al., 2011). Short stature has been reported in a number of patients (Hirano et al., 1994; Debouverie et al., 1997; Papadimitriou et al., 1998; Gamez et al., 2002; Martín et al., 2004; Garone et al., 2011). Furthermore, a case of erectile dysfunction has been diagnosed in a young male MNGIE patient (Schupbach et al., 2007).

### Histopathology

Skeletal muscle biopsy may show ragged-red fibers (due to abnormal proliferation of mitochondria in response to defective oxidative phosphorylation), ultra-structurally abnormal mitochondria, and abnormalities of both mtDNA and mitochondrial electron transport chain enzymes activities on enzyme analysis (Papadimitriou et al., 1998). However, it is important to note that ragged-red fibers are not always seen in MNGIE, as some patients do not display this histological abnormality (Szigeti et al., 2004b; Cardaioli et al., 2010).

Rectal biopsies show eosinophilic cytoplasmic inclusions in the submucosal ganglion cells (Perez-Atayde et al., 1998). Duodenal biopsies show focal muscle atrophy or absence, with increased nerve numbers, serosal granulomas and focal loss of Auerbach's plexus with fibrosis (Teitelbaum et al., 2002). Also, mtDNA depletion, mitochondrial proliferation and smooth cell atrophy are observed in the external layer of the muscularis propria in the stomach and small intestine (Giordano et al., 2006). Loss of interstitial cells of Cajal in the small bowel has also been reported (Zimmer et al., 2009; Yadak et al., 2018a).

Histopathological studies *post mortem* have failed to identify demyelination, neuronal loss or glial scarring in the areas of the brain white matter affected, as visualized by MRI (Szigeti et al., 2004a; Gramegna et al., 2018). However, the presence of albumin in the cytoplasm of reactive astrocytes was observed suggesting functional blood brain barrier alterations and consequent vasogenic oedema as a cause of leukoencephalopathy (Szigeti et al., 2004a). Furthermore, a mild perivascular gliosis was also observed in immunohistochemical analyses (Gramegna et al., 2018).

Ultra-structurally, peripheral nerve fibers show demyelination, and abnormal mitochondrial in Schwann cells (Hirano et al., 1994; Bedlack et al., 2004; Said et al., 2005). In addition to loss of myelinated fibers, nerve biopsies demonstrate mild perineural thickening, segmental demyelination, variation in internodal length and evidence of axonal regeneration (Hirano et al., 1994).

## Genotype-Phenotype Relationship

The primary clinical manifestations of MNGIE are well characterized and homogeneous (Cardaioli et al., 2010). However, one of the problematic aspects of MNGIE is that specific *TYMP* mutations do not necessarily correlate with distinct phenotypes, and therefore it is not possible to anticipate disease severity, system involvement and age of onset based on the mutation (Nishino et al., 2000). Indeed, individuals with the same *TYMP* mutation do not always exhibit the same phenotype, resulting in heterogeneity amongst patients. We hypothesize that clinical heterogeneity in MNGIE could be attributable to mtDNA heteroplasmy, as observed in other mitochondrial disorders (Morgan-Hughes and Hanna, 1999). For instance, siblings harboring the same mutations (435G>A) have been reported not to display an identical clinical phenotype, with the proband displaying both neurological and gastrointestinal symptoms, whereas the sibling had no gastrointestinal involvement (Gamez et al., 2002).

Some patients have been reported to manifest typical symptoms of MNGIE without any overt muscular abnormalities to confirm the diagnosis, suggesting that there might be a clear genotype-phenotype relationship in patients lacking skeletal muscle involvement (Szigeti et al., 2004b; Cardaioli et al., 2010).

It is unclear how each molecular variant affects the phenotype, however certain mutations have been associated with less severe enzyme dysfunction (10–15% residual activity), such as the 266G>A variant, which translates to milder manifestations and presentation of some of the canonical symptoms and a late onset of the disease (Marti et al., 2005; Massa et al., 2009).

Although the nervous and gastrointestinal systems are both affected, some patients display phenotypes characterized by a notably more prominent involvement of one or the other organ system (Gamez et al., 2002). The understanding of why one system is more affected than the other in certain patients remains unclear.

Furthermore, MNGIE-like manifestations occur in patients with normal thymidine phosphorylase activity, which are attributed to mutations in genes other than the *TYMP*, such as *POLG* and *RRM2B* (Nishino et al., 2001).

Heterozygotes for pathogenic *TYMP* mutations exhibit only 26–35% thymidine phosphorylase activity in buffy coats, which is sufficient to prevent the disease and the manifestation of a clear phenotype (Spinazzola et al., 2002).

## Diagnosis

### Diagnostic Challenges

The rarity of MNGIE and its multisystem nature contribute to a complex clinical picture that is often difficult for non-specialist healthcare professionals to decipher and provide an early diagnosis (Filosto et al., 2011). This can lead to diagnostic delays of between 5 and 10 years (Lara et al., 2007; Taanman et al., 2009). Although confirmation of the diagnosis by testing for thymidine and deoxyuridine in the urine and plasma, combined with Sanger sequencing of the *TYMP* gene is straightforward, initial identification of this rare condition often requires a clinical interdisciplinary approach, leading to diagnostic delays, and unnecessary invasive diagnostic procedures, such as exploratory surgeries for gastrointestinal disturbance or unnecessary treatments, such as intravenous immunoglobulin before the diagnosis is made. A late diagnosis is often associated with a worse prognosis (Scarpelli et al., 2012; Çoban et al., 2013). This situation advocates the urgent need for the early diagnosis of MNGIE. Thus, thymidine phosphorylase deficiency should be suspected in cases where gastrointestinal and neurological involvement coexist, particularly where there is leukoencephalopathy on MRI or abnormalities of ocular motility (Scarpelli et al., 2012). The symptoms of MNGIE often resemble other conditions which are usually included in the differential diagnosis. Frequently, patients are incorrectly diagnosed with anorexia nervosa, inflammatory bowel disease, Crohn's disease, Whipple disease, chronic intestinal pseudo-obstruction, coeliac disease, chronic inflammatory demyelinating polyneuropathy and demyelinating forms of Charcot-Marie-Tooth disease (Said et al., 2005; Needham et al., 2007; Filosto et al., 2011; Garone et al., 2011; Demaria et al., 2016; Imperatore et al., 2017; Nagata and Buckelew, 2017; Kucerová et al., 2018). Phenotypes resembling MNGIE may be seen in patients with other mitochondrial DNA depletion syndromes including *POLG* or *RRM2B* mutations and Kearns-Sayre syndrome. These are often referred to as pseudo-MNGIE manifestations (Shaibani et al., 2009; Garone et al., 2011; Prasun and Koeberl, 2014). More recently two cases of MNGIE-like patients exhibiting *POLG* mutations were reported to manifest leukoencephalopathy and demyelinating peripheral neuropathy, which are characteristic not typically observed with these mutations (Yasuda et al., 2018). Another case study, reports two patients with a MNGIE-like phenotype exhibiting optic atrophy associated with a novel *POLG* mutation affecting the C- terminal sub-domain of the protein (Felhi et al., 2018).

### Current Diagnostic Methods for MNGIE

#### Thymidine and Deoxyuridine Measurement in Plasma and Urine

Plasma thymidine and deoxyuridine levels are increased to >3 μmol/L and >5 μmol/L, respectively, compared to undetectable levels in healthy unaffected controls (Martí et al., 2003, 2004). Urine concentrations of thymidine and deoxyuridine are also increased (Spinazzola et al., 2002).

#### TP Activity

An evaluation of thymidine phosphorylase activity is typically required to complement the measurement of thymidine and deoxyuridine concentrations in body fluids, or upon the identification of novel variants of the *TYMP* gene, or when clinics do not have access to Sanger sequencing of *TYMP*. Thymidine phosphorylase activity in the leukocytes of patients with MNGIE are severely reduced, showing little (<10% of healthy unaffected controls) or no activity (Spinazzola et al., 2002; Martí et al., 2004). Heterozygous carriers of *TYMP* mutations have 26 to 35% of residual thymidine phosphorylase activity but are asymptomatic and have undetectable levels of plasma thymidine and deoxyuridine (Nishino et al., 1999; Spinazzola et al., 2002). These data suggest that a 70% reduction in thymidine phosphorylase activity is insufficient to be pathogenic.

#### Molecular Genetic Abnormalities

Patients are either homozygous or compound heterozygous for *TYMP* mutations and therefore the diagnosis is made by the detection of biallelic pathogenic variants in the gene (Nishino et al., 2001). For this reason genetic counseling is fundamental as the autosomal recessive inheritance translates to a 25% risk for offspring of carrier parents to be affected, whereas 50% will be asymptomatic carriers. Cases of MNGIE amongst twins have been reported, including a triplet in which two monozygotic pairs were affected whereas the dizygotic sibling was an asymptomatic carrier (Schupbach et al., 2007). Similarly, other case studies have described monozygotic twins carrying the same mutation and exhibiting the same phenotype (Papadimitriou et al., 1998; Bedlack et al., 2004). Genetic counseling should be made available to affected individuals and their families.

Targeted gene testing for primary *TYMP* mutations or more comprehensive genomic analyses for the whole genome including secondary mtDNA mutations can be used, such as Sanger or next generation sequencing, quantitative PCR, Southern blot, multiplex ligation-dependent probe amplification and genome-wide single nucleotide polymorphism microarrays (Katsanis and Katsanis, 2013). It is important to note that when biochemistry analyses are positive, revealing nucleoside accumulation and loss of thymidine phosphorylase function, Sanger sequencing is advisable. However, in case of doubtful biochemical profiling or negative detection of *TYMP* variants by Sanger sequencing, gene panels, whole exome sequencing (WES), whole genome sequencing (WGS) or mtDNA studies are recommended for the identification of MNGIE-like disorders.

Examination of mtDNA using Southern blot analysis has revealed abnormalities, including those which are quantitative (depletions) and qualitative (multiple deletions and point mutations) (Hirano et al., 1994; Papadimitriou et al., 1998; Nishino et al., 2000). An uneven distribution of mtDNA abnormalities (depletion, single nucleotide variants, deletions, duplication) along the nerves is hypothesized to be the cause of segmental demyelination. MtDNA depletion, mitochondrial proliferation, and smooth cell atrophy have been shown in the external layer of the muscularis propria in the stomach and small intestine (Giordano et al., 2006, 2008).

#### Clinical Examination

Currently the diagnosis is initially suspected based on clinical signs of gastrointestinal dysmotility, cachexia, peripheral neuropathy and ophthalmoplegia (Nishino et al., 2000; Garone et al., 2011). It is noteworthy that these clinical evaluations are not specific to the disease but are rather a general approach used for patients to assist in raising the suspicion of MNGIE. Audiologic, ophthalmologic evaluation and gastroenterology examinations such as abdominal CT, upper gastrointestinal tract contrast radiography, esophagogastroduodenoscopy, sigmoidoscopy, liquid phase scintigraphy and antroduodenal manometry are supportive for the diagnosis of MNGIE (Mueller et al., 1999; Teitelbaum et al., 2002; Halter et al., 2010).

#### MRI

Progressive and diffuse leukoencephalopathy is invariably observed in brain MRI of MNGIE patients, visualized as described above. Therefore, MRI is often used to evaluate one of the main clinical criteria of MNGIE. White matter MRI abnormalities provide a clear indication of the disease and in its absence MNGIE disease is very unlikely (Scarpelli et al., 2013). In fact, leukoencephalopathy helps discriminate between MNGIE and pseudo-MNGIE presentations of other disorders (Hirano et al., 2004b). However, a case study of patients with *POLG* mutations has been documented which present MNGIE-like phenotype exhibiting leukoencephalopathy on MRI, which is not a typical observation in these type of patients (Yasuda et al., 2018). One study also showed mild cortical atrophy and oculomotor and trigeminal nerve signal enhancement in T1 sequences (Petcharunpaisan and Castillo, 2010). Similarly, another study reported supratentorial cortical atrophy in patients with MNGIE (Barragán-Campos et al., 2005). Magnetic resonance spectroscopy (MRS) studies have also shown reduction in choline and N-acetyl aspartate indicating axonal loss and glial cells loss (Schupbach et al., 2007). However, in a recent study by Gramegna et al., MRS of patients with MNGIE has shown a consistent reduction of all metabolites in the white matter although their ratio to creatine remained in the normal range. This finding, combined with the increased radial water diffusivity in images, is suggestive of increases in water content which could be attributable to a possible increase in the BBB permeability rather than neural cell loss (Gramegna et al., 2018).

#### Electrodiagnostic Procedures

Electrodiagnostic procedures are valuable to confirm neuromuscular dysfunctions, which are one of the major clinical criteria for MNGIE. Neurogenic and myogenic abnormalities are commonly detected on electromyography. Nerve conductions studies typically show decrease in motor and sensory nerve conduction velocities and prolonged F-wave (Hirano et al., 2004b).

#### Biochemical Findings

Routine clinical biochemical studies do not provide specific clues to a diagnosis of for MNGIE, although these are helpful to corroborate features that are common in patients including lactic acidosis, indicative of an oxidative phosphorylation defect (Martí et al., 2004). Furthermore, mild elevation in serum lactic acid and serum pyruvate have been reported, as well as elevation in uric acid, lactate dehydrogenase and creatine kinase (Hirano et al., 1994; Teitelbaum et al., 2002). Increased levels of cerebrospinal fluid lactate and total protein have been described (Teitelbaum et al., 2002; Röeben et al., 2017). Severe hypokalaemia was also observed in two patients leading to muscle tetany and cardiac arrythmia (Garone et al., 2011).

## Pre-Clinical Experimental Models

*In vitro* and *in vivo* models of MNGIE have been developed to enhance the understanding of the disease pathogenesis and the development of experimental therapies, Table 2.

**Table 2:** *In vitro* and *in vivo* models of MNGIE.

**Cell type**	**Investigation**	**Summary of findings**	**References**
***In vitro*** **models**			
Healthy control and MNGIE fibroblasts	Contribution of thymidine phosphorylase deficiency to nucleotide pool imbalance	Decline in thymidine concentration in culture medium of healthy cells. MNGIE fibroblasts incapable of metabolising thymidine but released it	Spinazzola et al., 2002
MNGIE fibroblasts	Role of thymidine phosphorylase deficiency and deoxynucleotide pool accumulation in mtDNA damage	Identification of 36 mtDNA point mutations, a TT to AA substitution and single nucleotide deletion in MNGIE cell lines. COX activity reduced and ROS production increased contributing to mtDNA mutations	Nishigaki et al., 2003
HeLa cell line	Perturbation of deoxynucleoside pools in cultured cells to evaluate mtDNA damage	Cells cultured in 50μM thymidine showed expansion of TTP and dGTP pools and depletion of dCTP and dATP pools. Several mtDNA deletions observed	Song et al., 2003
Healthy skin and lung quiescent fibroblasts	Association of mtDNA depletions with post-mitotic cells	Thymidine phosphorylated via mitochondrial TK2 in quiescent cells and via cytosolic TK1 in cycling cells. Absence of TK1 in quiescent creates a bias in TTP pools, contributing to mtDNA depletions	Ferraro et al., 2005
Murine hepatocytes	Murine hepatocyte mitochondria as an *in organello* model to demonstrate mtDNA depletion is a result of deoxynucleoside depletion	Excess thymidine resulted in increased dTTP and consequent depletion of dCTP, due to competition of thymidine and cytidine for TK2, resulting in mtDNA depletion. Supplementation of dCTP restored mtDNA depletions	González-Vioque et al., 2011
MNGIE-derived iPSCs	Differentiation of patient derived iPSCs into cerebral organoids as an *in vitro* model of the CNS	MNGIE cerebral organoids expressed neuronal progenitors, neurons, differentiated astroglial cells and myelinating oligodendrocytes. No difference in myelination patterns observed between MNGIE and healthy control organoids	Pacitti and Bax, in press
***In vivo*** **models**			
Murine KO (*Tymp^−/−^/Upp1^−/−^*)	Physiological function of thymidine phosphorylase. Ascertain if pathogenesis of MNGIE and mtDNA depletion and replication error were attributable to aberrant thymidine metabolism	10-fold increase in plasma deoxyuridine and thymidine. Development of cerebral oedema and hyperintense T2 MRI regions, with dilation in axonal myelin fibers but no demyelination. No peripheral neuropathy observed. Lack of mtDNA abnormality in brain and muscle	Haraguchi et al., 2002
Murine KO (*Tymp^−/−^/Upp1^−/−^*)	Characterization of the biochemical, genetic and histological features of MNGIE and specific tissues involved	Undetectable thymidine phosphorylase in all tissue except liver. Thymidine elevated by 4-65-fold in all tissues. MRI showed cerebral oedema and T2 hyperintensities, with late onset cerebral and cerebellar white matter vacuoles without demyelination or axonal loss. Detection of mtDNA depletion and histological abnormalities in the brain but without skeletal muscle and gastrointestinal system involvement	López et al., 2009
Murine KO (*Tymp^−/−^/Upp1^−/−^*)	Role of deoxynucleoside accumulation in the pathogenesis of MNGIE. Recreation of the gastrointestinal phenotype by dietary supplementation with thymidine and deoxyuridine	100-fold increase in thymidine concentrations. Acquisition of mtDNA depletion and histologically evident COX deficiency in brain and small intestine cells. Treated mice had reduced body masses and intestinal smooth muscle cells, and increased fibrosis, muscle weakness, leukoencephalopathy, and decreased survival	Garcia-Diaz et al., 2014

### *In vitro* Models

There is a paucity of specific *in vitro* models of MNGIE described in the literature. A majority of the cell types implemented to date have no relevance to the organ systems affected in MNGIE and were used mainly to understand the effect of deoxyribonucleoside pool imbalances on cellular functions (Rampazzo et al., 2000, 2004; Pontarin et al., 2003). The first model developed was by Spinazzola et al. (2002), where fibroblasts derived from healthy controls and patients with MNGIE were used to study the contribution of thymidine phosphorylase in the deoxyribonucleoside pool imbalances. They examined the culture medium of cultured fibroblasts to determine the ability of healthy control cells and MNGIE patient cells to metabolize thymidine; in contrast to healthy cells, where a decline in media thymidine concentrations was measured, MNGIE fibroblasts were not able to catabolize thymidine, resulting in an increase in culture medium thymidine levels (Spinazzola et al., 2002).

Nishigaki et al. (2003) employed MNGIE-derived fibroblast to further evaluate the role of dysfunctional thymidine phosphorylase in the accumulation of deoxyribonucleoside pools (both thymidine and deoxyuridine) and with consequent mtDNA damage. Using patient-derived cell lines, 36 mtDNA point mutations, a TT to AA substitution and a single nucleotide deletion were identified. In MNGIE fibroblast cultures, cyclooxygenase activity was decreased, whereas deoxyuridine levels were markedly elevated. Also, an elevation in reactive oxygen species was observed, which was proposed to be a contributing factor to the accumulation of mtDNA point mutations. MtDNA sequencing of cultured fibroblasts and *post mortem* biopsies of skeletal muscle cells revealed a higher level of mtDNA point mutations in fibroblasts, whereas multiple mutations and deletions were observed at low levels in skeletal muscles. This suggests that fibroblasts primarily depend on anaerobic glycolysis rather than oxidative phosphorylation and therefore the absence of pressures on defective respiratory chain complexes in mitochondria results in the accumulation of nucleotide pools that generates a higher number of point mutations (Nishigaki et al., 2003).

In 2003, Song et al. used HeLa cells to show that increases in thymidine levels lead to an imbalance in dNTP pools, which ultimately result in mtDNA mutations. HeLa cells were cultured in medium supplemented with 50 μM thymidine. After 4 h of growth in thymidine-supplemented medium, the mitochondrial deoxythymidine triphosphate (dTTP) and deoxyguanosine triphosphate (dGTP) pools were shown to expand, whereas the deoxycytidine triphosphate (dCTP) pool dropped significantly, and the dATP pool dropped slightly. In whole cell extracts, the dTTP and dGTP pools also expanded, the dCTP pool decreased by approximately 50%, and the dATP pool remained unchanged. These changes in mitochondrial dNTP pools are consistent with a mutagenic mechanism involving the T-G mispairing followed by a next-nucleotide effect involving T insertion opposite to A. Supplementation of HeLa cells for 8 months with 50 μM thymidine, resulted in several mtDNA deletions (Song et al., 2003). It is noteworthy that to recreate MNGIE metabolite accumulations, the study implemented a 2.5-fold higher thymidine concentration to that observed in MNGIE patients, which is typically 20 μM thymidine (Song et al., 2003; Ferraro et al., 2005).

In 2005, Ferraro et al. conducted a similar experiment to Spinazzola et al. (2002), but used healthy skin and lung quiescent fibroblasts to demonstrate that mtDNA depletions are associated with post-mitotic cells. The study identified that mitochondrial deoxynucleotides are synthesized by two independent salvage pathways. In cycling cells, thymidine is salvaged by cytosolic thymidine kinase 1 (TK1) whereas in quiescent cells, thymidine is phosphorylated via thymidine kinase 2 (TK2) in the mitochondria, and the thymidine diphosphates then exported to the cytosol. Both cytosolic and mitochondrial thymidine phosphates undergo rapid turnover via deoxythymidine monophosphate (dTMP)/ thymidine substrate cycles. Therefore, quiescent cells lacking *de novo* synthesis and TK1 create a bias in dTTP pools, and this is further exacerbated in MNGIE where thymidine phosphorylase is lacking. Ferraro et al. (2005) cultured quiescent fibroblasts in medium supplemented with 10–40 μM thymidine and observed intra-cytosolic and intra-mitochondrial increase in dTTP and uridine triphosphates, both contributing to mtDNA depletions, concluding that mitochondrial DNA damage in MNGIE is predominant in post-mitotic cells (Ferraro et al., 2005).

González-Vioque et al. (2011), made use of murine liver mitochondria to show that mtDNA depletions are a consequence of a limited dNTP availability rather than a dNTP imbalance itself. The study demonstrated that excess of thymidine results in an increase of dTTP concentrations in mitochondria due to TK2 activity, with consequent secondary depletion of dCTP. TK2 phosphorylates both thymidine and cytidine competitively; each deoxynucleotide modulates the enzyme to consequently inhibit the phosphorylation of the other, although thymidine is more efficient at inhibiting the phosphorylation of cytidine. The addition of dCTP or deoxycytidine restored mtDNA depletions even in the presence of thymidine overload, confirming that mtDNA depletions are the result of a limited availability of substrates for mtDNA replication, caused by nucleotide depletion consequent to nucleotide overload rather than thymidine excess alone (González-Vioque et al., 2011).

Overall, *in vitro* models developed so far, have been informative and relevant for the understanding of the underlying biochemical and molecular mechanisms, associated with the deoxyribonucleoside pool imbalances and mtDNA depletions. However, to date tissue-specific models using cells relevant to the CNS, PNS and enteric system have not been developed and thus the study of alternative implications for the lack of thymidine phosphorylase expression on other biological pathways, including nervous tissue development and maintenance, has not been fully addressed.

Our research group has developed for the first time a MNGIE iPSC line which was used to generate a cerebral organoid model for the study of the CNS pathomolecular mechanisms and provide elucidations on the leukoencephalopathy observed (Pacitti, 2018; Pacitti and Bax, in press).

### *In vivo* Models

Murine models have proved to be very efficacious in the study of MNGIE, though it is important to consider the significant biological and hence metabolic differences between rodents and humans. This is exemplified by the metabolism of thymidine in the mouse, which is not only phosphorylated by thymidine phosphorylase, but also by uridine phosphorylase 1 and uridine phosphorylase 2; in the human, thymidine is solely metabolized by thymidine phosphorylase (el Kouni et al., 1993). To address this, Haraguchi et al. (2002) established a murine model based on double knock-out of *Tymp*^−/−^*/Upp1*^−/−^ genes, whilst uridine phosphorylase 2 is not knocked-out in this model. Although this model recapitulates some features of the disease, it also displays some incongruences with the clinical scenario. The knock-out animals have a 10-fold increase in plasma thymidine and deoxyuridine, compared to >100-fold increase in the human. The mice also show cerebral oedema with hyperintense T2 MRI regions and axonal myelin fiber dilation without demyelination, however no peripheral neurological abnormalities were observed (Haraguchi et al., 2002). Also, Haraguchi et al. (2002) did not detect any mtDNA abnormalities in brain and muscle tissues of mice, suggesting that the loss of function of thymidine phosphorylase alone is not sufficient to cause MNGIE in this model. This led to the hypothesis that the adjacent gene *SCO2* overlapping with *TYMP* sequences may be also contributing to the disease. The lack of mtDNA depletion in mice may be the result of a difference in mtDNA repair and replication, by which an increase in thymidine concentration may not affect the mitochondria of mice as it does in humans (Haraguchi et al., 2002).

A second double knock-out murine model of MNGIE was created in 2009 by Lopez et al. to characterize the biochemical, genetic and histological features of MNGIE in mice, and translate findings into the clinical picture (López et al., 2009). The resulting mice displayed undetectable thymidine phosphorylase activity in all tissues except in the liver, where the residual 17% activity was attributed to the expression of uridine phosphorylase 2. Mice displayed a 4- to 65-fold increase in thymidine levels in all tissues, with partial mtDNA depletions. Similarly, to the model developed by Haraguchi et al. (2002), the rodents manifested cerebral oedema with hyperintense T2 MRI signals in white matter, and late-onset cerebral and cerebellar white matter vacuoles without demyelination or axonal loss. However, in contrast to MNGIE patients, the model displayed mtDNA depletion, respiratory chain defects and histological abnormalities only in the brains, without any gastrointestinal or skeletal muscle involvement. López et al. (2009) suggested that the selective cerebral involvement observed in mice is possibly due to a number of factors, including differences in the life-span between species, as mice may not live long enough to accumulate sufficient mtDNA damage in most tissues or because the deoxyribonucleoside imbalance in humans is substantially more dramatic than in mutant mice. A third explanation is that high expression of TK2 in quiescent neuronal cells of rodent brains may contribute to an increased TTP production, thereby accelerating mtDNA damage in nervous tissues (Rylova et al., 2007). With regard to the contrasting findings of mtDNA depletions between Haraguchi's and Lopez's model, this could be explained by limitations in the analytical methods used for evaluating mtDNA aberrations (López et al., 2009).

In 2014, Garcia-Diaz et al. conducted a study to confirm the hypotheses generated by López et al. (2009) with regard to the role of thymidine accumulation in the pathogenesis of MNGIE, and in particular in the gastrointestinal involvement. López et al. (2009) failed to recapitulate the gastrointestinal dysmotility in mutant mice, and only replicated certain pathological features in mouse brains. It was speculated that the mild phenotype observed in the model is attributable to the short life-span of the animals combined with the modest increase in deoxyribonucleoside accumulation produced by mutant mice (which was 45-fold lower than that observed in MNGIE patients). Thus, to overcome this, Garcia-Diaz et al. (2014) supplemented mutant mice with exogenous thymidine and deoxyuridine to recreate a similar disproportion of deoxyribonucleoside concentrations as observed in humans, recapitulating the >100-fold increase in thymidine concentrations. The prolonged supplementation of deoxyribonucleosides in mutant mice resulted in the acquisition of biochemical abnormalities in the brain and small intestine, including mitochondrial DNA depletion as evidenced by cyclooxygenase deficiency observed through histological evaluations. Overall, treating double knock-out mice with thymidine was sufficient to enhance the phenotype of the model to recapitulate the clinical features of MNGIE, including weight loss, small intestine muscularis propria pathology, muscle weakness, leukoencephalopathy and decreased survival (Garcia-Diaz et al., 2014). However, in contrast to patients with MNGIE, who have multiple mtDNA deletions in brain, muscles, kidney and liver, the brain and muscle of 24-month old treated and untreated wild-type and *Tymp*^−/−^*/Upp1*^−/−^ mice demonstrated similar levels of deleted mtDNA, suggesting that this is most likely due to aging rather than thymidine phosphorylase deficiency (Garcia-Diaz et al., 2014). Differences in the deoxyribonucleoside metabolism between humans and mice indicates the inadequacy of this model in recapitulating the human disease (Haraguchi et al., 2002; López et al., 2009; Garcia-Diaz et al., 2014).

## Treatment Options

### Disease Management

Currently, there are no specific therapies for patients with MNGIE whose effectiveness has been evidenced in clinical trial studies. The current disease management guidelines aims to treat the specific symptoms that are evident in each individual and invariably requires the co-ordinated effort of different clinical specialities. Abdominal pain and nausea/vomiting secondary to gastrointestinal dysmotility are almost invariable, with patients treated symptomatically with analgesics, bowel motility stimulant drugs, anti-emetics and antibiotics for intestinal bacterial overgrowth (Teitelbaum et al., 2002; Oztas et al., 2010). Domperidone may be administered to control the post-prandial emesis and nausea (Yavuz et al., 2007). The reduction of epigastric pain episodes, especially in patients that are refractory to opiate pain management, can be achieved by performing a celiac plexus block with bupivacaine or by the selective blockade of the splanchnic nerve (Teitelbaum et al., 2002; Celebi et al., 2006). Pain may also occur in the limbs due to peripheral polyneuropathy and this can be treated with centrally acting agents such as amitriptyline, gabapentin and pregabalin (Hafez et al., 2014; Finsterer and Frank, 2017). Patients with MNGIE have an increased incidence of perforation of the gut, which generally requires emergency abdominal surgery (Granero Castro et al., 2010).

Malnutrition is a major problem in the majority of patients; various forms of parenteral nutrition, including total parenteral nutrition, are frequently required, but do not modify outcome (Wang et al., 2015). Complications of long-term parenteral nutrition use include the development of hepatic steatosis and cholestasis, and triglyceride hyperlipidemia. For patients with MNGIE there is the risk of metabolic oversupply from the lipid and carbohydrate components of the parenteral nutrition, leading to further mitochondrial toxicity. In later stages of the disease, patients are often unable to tolerate nasogastric nutrition due to gastrointestinal dysmotility (Wang et al., 2015). Portal hypertension may occur and be complicated by ascites and esophageal varices (Moran et al., 2008). These conditions are treated in the same way as when they occur in other conditions. Drugs that interfere with mitochondrial function should be avoided and hepatically metabolized drugs should be administered with care or contraindicated depending on the patient's liver function (Halter et al., 2010). Physiotherapy and occupational therapy input is usually required, particularly to address the neurological aspects of the condition.

### Investigational Therapies

A number of experimental therapeutic approaches are currently under investigation, including haemodialysis and peritoneal dialysis (Spinazzola et al., 2002; la Marca et al., 2006; Yavuz et al., 2007), allogeneic haematopoietic stem cell transplantation (AHSCT) (Hirano et al., 2006; Halter et al., 2010; Filosto et al., 2012), platelet transfusion (Lara et al., 2006), orthotopic liver transplant (OLT) (De Giorgio et al., 2016) and enzyme replacement (Bax et al., 2013). The therapeutic strategy common to all these approaches is to reduce or eliminate the pathological concentrations of thymidine and deoxyuridine, thereby ameliorating intracellular deoxyribonucleoside imbalances and preventing further damage to mtDNA, thus translating into clinical stabilization or improvement.

Plasma concentrations of thymidine were shown to be transiently lowered by haemodialysis, and infusions of platelets, which contain thymidine phosphorylase, were shown to reduce circulating levels of thymidine and deoxyuridine in two patients (Lara et al., 2006; Röeben et al., 2017). Disadvantages of these approaches are that haemodialysis is a burdensome procedure and long-term platelet therapy carries risks of developing immune reactions and transmission of viral infections and the short duration of effect.

AHSCT offers the possibility of a permanent correction of the thymidine phosphorylase deficiency but is limited by the availability of a matched donor. Patients are often in a poor clinical condition with an impaired capacity to tolerate transplant related problems and the aggressive conditioning and immunosuppressive chemotherapy (Halter et al., 2010, 2015). AHSCT also presents pharmacological challenges in terms of administering drugs with possible mitochondrial toxicity, and the requirement for parenteral administration due to disturbed gastrointestinal function and impairment of absorption. A published consensus proposal for standardizing an approach to AHSCT in patients with MNGIE recommended a recruitment restriction to patients in a stable clinical condition without irreversible end stage disease and having optimal donor (Halter et al., 2010). AHSCT is associated with an elevated mortality risk due to host-vs. -graft reactions and hospital acquired infections caused by the aggressive immunosuppressive regimen, combined with the disease (Halter et al., 2010; Filosto et al., 2012; Peedikayil et al., 2015). Halter et al. (2015) reported a mortality of 62.5% after the follow-up of 24 patients who received AHSCT (Halter et al., 2015). Patients who are oligosymptomatic are often reluctant to undergo AHSCT due to its high morbidity and mortality risk. A recent study suggested that the effect of AHSCT may be transient (Baker et al., 2017). Furthermore, a study focusing on the neuromuscular pathology of the small intestine in MNGIE highlighted that AHSCT may be insufficient to restore integrity of the enteric neurons and glia, thus without any short-term impact on the neurogenic and myogenic intestinal changes observed in later stages of MNGIE (Yadak et al., 2018a).

Due to the elevated expression of thymidine phosphorylase in the liver, solid organ transplantation is considered an alternative long-term therapeutic option (Boschetti et al., 2014). A case study has shown that OLT was able to normalize metabolite levels and provide mild improvements of neurological symptoms (De Giorgio et al., 2016; D'Angelo et al., 2017). The extent to which tissue damage can be reversed through the clearance of deoxyribonucleoside imbalances post- OLT has yet to be determined (De Giorgio et al., 2016).

**Figure 6:**
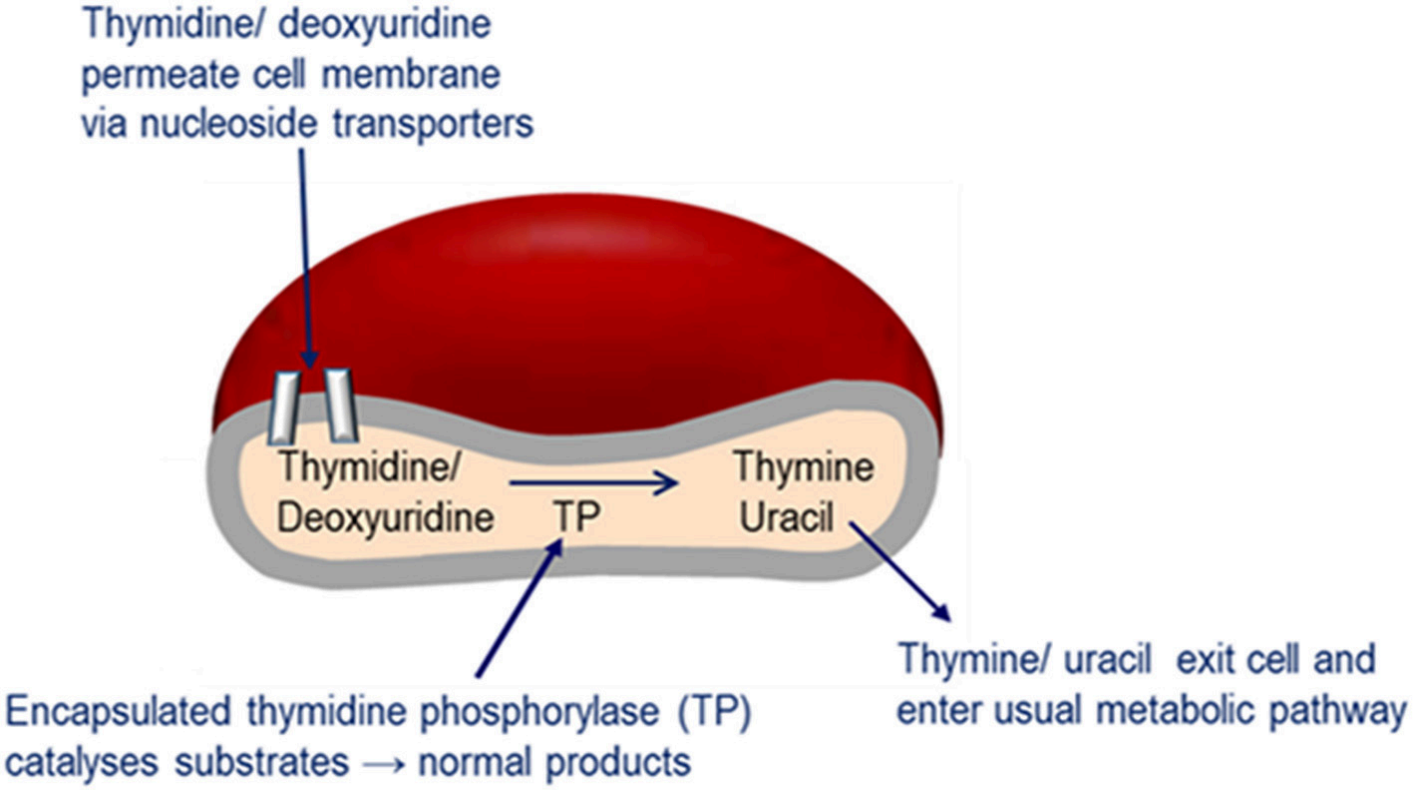
Mechanism of EE-TP action. Plasma thymidine and deoxyuridine enter the erythrocyte via nucleoside transports located in the cell membrane, where the encapsulated thymidine phosphorylase catalyses their metabolism to thymine and uracil. The products are then free to diffuse out of the cell into the blood plasma where they can enter the normal metabolic pathways.

Enzyme replacement therapy using autologous erythrocyte-encapsulated thymidine phosphorylase (EETP) is under investigation and has Orphan drug Designation by the FDA and EMA. The rationale for the development of EETP is based on thymidine and deoxyuridine being able to freely diffuse across the erythrocyte membrane via nucleoside transporters into the cell where the encapsulated enzyme catalyses their metabolism to the normal products (Figure 6). The products are then free to exit the cell into the blood plasma where they are further metabolized as normal. EETP is directed at ameliorating thymidine and deoxyuridine levels to slow the progression of MNGIE and stabilize the clinical condition and could therefore increase the chance of eligibility for AHSCT or OLT once a match is identified. Encapsulation of enzyme within the erythrocyte has the pharmacological advantages of prolonging the circulatory half-life of the enzyme and potentially minimizing immunogenic reactions which are frequently observed in enzyme replacement therapies administered by the conventional route. To date five patients have received EETP under a compassionate use programme, where clinical and metabolic improvements were observed (Moran et al., 2008; Halter et al., 2010; Godfrin and Bax, 2012; Godfrin et al., 2012; Bax et al., 2013).

**Figure 7:**
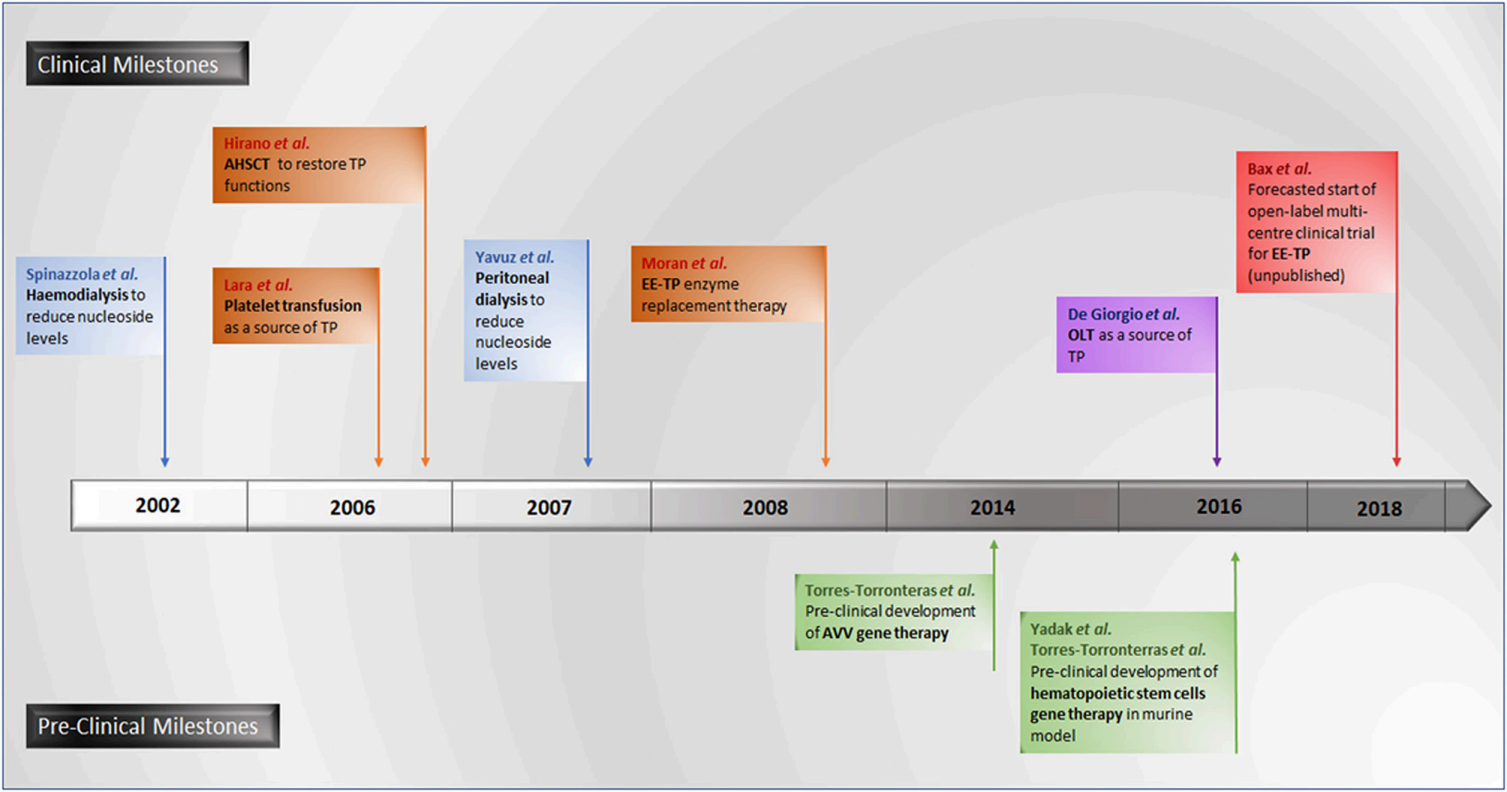
Timeline of pre-clinical and clinical investigational therapeutic approaches for MNGIE.

Promising gene therapies for MNGIE are also under experimentation in murine models, using adenoviral vectors (AVV) targeting the liver for the correction of *TYMP* mutations for the restoration of normalized nucleoside metabolism (Torres-Torronteras et al., 2014). More recently, pre-clinical investigations of hematopoietic stem cell gene therapy in murine models have been conducted (Torres-Torronteras et al., 2016; Yadak et al., 2018a,b). A timeline of all investigational therapeutic approaches is summarized in Figure 7.

### Clinical Efficacy Endpoints

The development of drugs for rare diseases is confounded by a number of challenges such as small patient populations, phenotypic heterogeneity, incomplete knowledge of the disease pathophysiology or natural history and an absence of prior clinical studies. Consequently, the selection of clinical efficacy endpoints, which assess the way a patient feels, functions, or survives, can be an arduous process, particularly as validated endpoints appropriate for the disease are often unavailable.

There are generally no accepted endpoints for clinical studies in patients with MNGIE. This ultra-rare disease presents with usually a combination of cachexia, gastrointestinal dysfunction, and neuromuscular dysfunction. The determinants of morbidity and mortality in patients with MNGIE cannot be easily ascertained and owing to the rarity of the disease, there is no authoritative literature on the topic. The available case series of patients with MNGIE are small and with limited follow-up; the heterogeneity of the sources further limits the possibility to collate this information objectively. Additionally, there are no established patient reported outcomes specific to MNGIE. The experimental treatments for MNGIE aim to reverse the biochemical imbalances by eliminating the elevated systemic concentrations of thymidine and deoxyuridine. However, these metabolites do not provide objective measurements correlated to clinical status and are thus not suitable as end-points for predicting clinical benefit of therapeutic strategies. Several patients reported outcomes are available for specific symptoms or groups of symptoms (e.g., gastrointestinal, neuropathic) which are highly prevalent in patients with MNGIE; however, the extent to which those measurement instruments would be applicable to patients with MNGIE is unknown.

Despite genotypic differences and a variable phenotype, gastrointestinal symptoms including early satiety, nausea, dysphagia, gastroesophageal reflux, postprandial emesis, episodic abdominal pain, episodic abdominal distention, and diarrhea are cardinal manifestations of MNGIE and severely compromise nutritional homeostasis in almost all patients, leading to weight loss and cachexia. One of the largest case series available reports a “thin” body habitus in all patients, with weight loss from diagnosis averaging 15.2 kg (range: 5.9–30.0 kg) (Nishino et al., 1999).

Although clinicians treating patients with MNGIE, unanimously agree that weight loss is the key feature of the disease and has a major impact on their functional status, individual weight loss trajectories are not typically available in published case series. The consensus in personal communications with clinicians who treat those patients suggests that patients with MNGIE relentlessly lose weight and that this has a major impact on their functional status. Anecdotal evidence based on a review of case series and case studies in the literature suggests that organ failure, hepatic and gastrointestinal complications associated to cachexia are frequent causes of death in patients with MNGIE (Nishino et al., 2000; Garone et al., 2011).

The collection of uniform observational data through the operation of patient registries is one approach that is employed to identify suitable efficacy endpoints. Registries are particularly relevant to the field of rare diseases where the disorder has a heterogeneous presentation and information on the natural history is scarce. Of relevance to patients with MNGIE is the Rare Disease Clinical Research Network Natural History Study of MNGIE (NCT01694953) and also the North American Mitochondrial Disease Consortium which is currently collecting medical and family history, diagnostic test results, and prospective medical information; information from this will be invaluable for supporting the evaluation of new treatment modalities.

The Regulatory agencies are now recognizing the need for flexibility in the review of therapies for rare diseases and may consider approving a therapy based on a surrogate endpoint or biomarker as these can provide better objective measures of clinical benefit. Based on the identification of a number of dysregulated miRNAs in the serum of patients with MNGIE compared to age and sex matched healthy controls, Levene et al. are examining the application of a miRNA panel as a surrogate end-point biomarker in parallel with a clinical trial of EETP (Levene et al., in press).

## Prognosis

MNGIE is a relentlessly progressive degenerative and terminal disorder with a poor prognosis. The estimated mean age of mortality is 37.6 years, with a range of 26–58 years (Nishino et al., 2000). Garone et al. reports the use of a Kaplan-Meier analysis, as a valuable instrument to give a reliable prognosis, thus providing the most updated estimates in term of life expectancy to date, indicating that in MNGIE survival lies between 20 and 40 years of age. Common causes of death include malnutrition, metabolic acidosis, aspiration pneumonia, intestinal perforation, peritonitis and complications aroused by bacterial overgrowth (Filosto et al., 2011; Garone et al., 2011).

## External Resources for Clinicians and Patients

Below we present a list of resources for clinicians and patients:

https://www.omim.org/entry/603041
https://rarediseases.org/rare-diseases/mitochondrial-neurogastrointestinal-encephalopathy/
https://clinicaltrials.gov/ct2/show/NCT01694953
https://www.mitocon.it/classificazione-genetica-delle-malattie-mitocondriali/
https://ghr.nlm.nih.gov/condition/mitochondrial-neurogastrointestinal-encephalopathy-disease
https://www.orpha.net/
www.telethon.it
http://www.pumpa.org.uk/
https://www.thelilyfoundation.org.uk/
http://www.umdf.org/
http://www.mitoaction.org/

## Concluding Remarks

MNGIE is a metabolic disorder with an invariably fatal outcome. In the last 40 years since the first description of MNGIE, considerable progress has been made in the elucidation of the pathogenic mechanisms that underlie this ultra-rare disease. The wealth of knowledge available enabled the canonical and the sporadical features of the pathology to be clearly defined, permitting explicit diagnostic criteria and approaches to be determined. Recently, Zimmer et al. proposed an algorithm for the clinical diagnosis of MNGIE, whereby patients exhibiting suggestive clinical symptoms, such as eye movement disorder, are recommended to undergo cerebral MRI investigations. If leukoencephalopathy is then detected, the algorithm suggests biochemical testing for metabolite excess in biofluids, followed by the sequencing of the *TYMP* gene, should an accumulation of thymidine and deoxyuridine be identified (Zimmer et al., 2014). It is important to highlight however, that MNGIE, as for many other mitochondrial disorders lacks of a prospective natural history study, although one is currently ongoing and pending results. In this respect patient stratification, still remains a substantial challenge. Nevertheless, the advent of NGS, has certainly changed the diagnostic approach toward mitochondrial diseases, including MNGIE, thus reliably improving the screening and clustering of patients. Therefore, in many cases a shift in diagnostic methodologies is observed toward a direct genetic screening (Calvo et al., 2012; Carroll et al., 2014; Craven et al., 2017; Kremer et al., 2017). On the other hand, NGS has not entirely replaced the use of the first line investigations for the identification of MNGIE, i.e., quantifying thymidine and deoxyuridine in plasma and urine. In fact, recent research efforts have been directed at improving the analytical methods used. For instance, optimized and validated methods aimed at simplifying the chromatographic conditions and reducing analytical errors for the quantification of thymidine and deoxyuridine in urine and plasma of MNGIE patients, was developed and compared with previously reported analytical methods (Mohamed et al., 2014; Sun, 2016). It is noteworthy that advancements in experimental therapies for MNGIE are mostly of recent development; indeed, the first published data collection of all patients treated with AHSCT dates back to 2015, sixteen years after the mutation was first identified (Halter et al., 2015). It is also important to note, that predominantly in eastern countries, the most dated therapeutic approach, more specifically peritoneal dialysis and haemodialysis, are still being used in the management of MNGIE (Sivadasan et al., 2016; Chandra et al., 2018). A number of experimental therapies are currently under development with the aim of rescuing the phenotype by restoring homeostatic thymidine phosphorylase activity and/or normalizing systemic deoxyribonucleoside accumulations. Most notably, a recent study conducted by Torres-Torronteras et al., describes a novel promising pre-clinical investigation regarding the long-term efficacy of AVV gene therapy in MNGIE (Torres-Torronteras et al., 2016). However, there is still a substantial gap between pre-clinical trials and the translation of novel treatments into humans. Furthermore, the rarity of the condition and the absence of a natural history study hinders the identification of reliable end-points, further complicating the progression of experimental therapies. In this respect, MNGIE benefits from clinical interest because is one of the few treatable rare mitochondrial disorders (Filosto et al., 2018). With the up and coming clinical trials of these novel therapeutic approaches, including the enzyme replacement therapy under investigation by our group, we believe this comprehensive review will guide and inform clinicians of the intricacies of this rare and fatal disorder, thereby expediting disease diagnosis and treatment access to patients earlier on in the disease process.

## Author Contributions

DP, ML, CG, NN, and BB contributed to the conception, writing and review of the manuscript.

### Conflict of Interest Statement

St George's, University of London holds a licencing agreement with Orphan Technologies for the development of an enzyme replacement therapy for MNGIE. The authors declare that the research was conducted in the absence of any commercial or financial relationships that could be construed as a potential conflict of interest.
